# Exploring menstrual health knowledge, attitude, and experiences among adolescent girls in public schools in Georgia: a qualitative study

**DOI:** 10.1186/s12978-026-02417-7

**Published:** 2026-07-25

**Authors:** Esma Imerlishvili, Elene Kaikhosroshvili, Ani Beraia, Mariam Beridze, David Otiashvili

**Affiliations:** 1 Association for Well-being of Society, Mtskheta, Georgia; 2 Youth for Public Health, Tbilisi, Georgia; 3 School of Natural Sciences and Medicine, Ilia State University, Tbilisi, Georgia

**Keywords:** Menstrual health, Public school, Georgia, Qualitative research, SEM, School health, Gender equity

## Abstract

**Background:**

Despite being a physiological process for human development, menstruation can be burdened with a complex interplay of interpersonal, social, and structural factors. Evidence on menstrual health remains scarce across Eastern Europe and Central Asia, and published research specifically addressing adolescent girls’ menstrual health experiences in Georgia is lacking, despite cultural and historical factors that suggest unique challenges in this setting. Public schools represent a critical but understudied environment where infrastructural, educational, and social barriers to menstrual health converge for adolescent girls. This study aims to explore menstrual health knowledge, attitudes, and experiences among schoolgirls in Georgia.

**Methods:**

We conducted a qualitative study using in-depth interviews and focus group discussions in public schools across urban and rural areas of three cities in Georgia in 2020. An inductive-deductive approach was used for data analysis. We used the Social Ecological Model (SEM) to report and interpret the findings.

**Results:**

Nine teachers and 39 schoolgirls (28 in six focus groups, 11 in interviews) participated. The Social Ecological Model revealed multilevel challenges: knowledge gaps and misconceptions (individual level); hindered family communication and peer stigma (interpersonal level); inadequate school infrastructure and insufficient teacher training (organizational level); cultural taboos shaping attitudes and practices (community level); and curriculum deficiencies preventing comprehensive education (policy level). These interconnected factors negatively affected schoolgirls’ menstrual health knowledge, hygiene management, and participation in school activities.

**Conclusion:**

The findings demonstrate that formal educational settings in Georgia inadequately address the schoolgirls’ menstrual health needs. Comprehensive menstrual health education, improved school infrastructure, and teacher training are essential to reduce menstrual stigma and provide accurate information and resources. Addressing these multilevel barriers can significantly enhance menstrual health, educational participation, and overall well-being among schoolgirls across Georgia.

**Supplementary Information:**

The online version contains supplementary material available at https://doi.org/10.1186/s12978-026-02417-7.

## Introduction

Menstrual health is defined as complete physical, mental, and social well-being in relation to the menstrual cycle [1]. It is increasingly recognized as a critical public health and human rights issue [2]. Approximately 1.9 billion individuals worldwide menstruate, yet an estimated 500 million are unable to attain adequate menstrual health [3]. Unmet menstrual health needs significantly affect school performance, participation, and overall well-being among adolescent girls [4–6]. Studies in sub-Saharan Africa found that one in ten girls missed school during menstruation, while research in Ethiopia documented that 50% of girls missed between 1 and 4 days monthly [7, 8]. Even in high-income settings such as the United Kingdom, 64% of girls aged 14–21 missed part or all of a school day due to their period [9]. These disruptions to education have long-term implications for the Sustainable Development Goals (SDG), particularly in relation to health, gender equality, and educational attainment [10].

Schools can promote adolescent health and well-being through curriculum content, supportive school environments, and engagement with families and communities [11]. However, many teachers report discomfort discussing menstruation, limited training, and a lack of awareness of menstrual stigma as a barrier to academic participation [12]. Adolescent girls worldwide face multiple menstrual health challenges, including limited access to menstrual products, inadequate water, sanitation, and hygiene (WASH) facilities, stigma, misinformation, and cultural taboos [13, 14]. Only 31% of schools globally have bins for menstrual waste in girls’ toilets [15]. In school settings, inadequate sanitation facilities, lack of privacy, and fear of teasing by peers are key barriers to school attendance and participation during menstruation [16, 17]. In many contexts, girls typically receive menstrual information from their family members and peers; this knowledge is often inadequate, inaccurate, and not evidence-based [18]. As a result, a substantial proportion of adolescent girls are unable to identify menstrual health problems or manage menstruation with dignity [19, 20]. Worldwide, only 39% of schools provide menstrual health education [15]. Cultural norms perpetuate menstrual stigma across generations, negatively affecting health, well-being, and self-esteem [21].

Although scientific literature is extremely scarce in Eastern Europe and Central Asia (EECA) [22], technical reports, together with the cultural, historical, and political context, suggest that menstrual health is particularly challenging, especially in school settings. Most territories in the region are former Soviet Union countries, where reproductive and sexual health education was systematically suppressed, with such topics treated as taboo and excluded from public discourse [23]. In the EECA region, comprehensive sexuality education, which often encompasses menstrual health, is mandatory in only 11 of 21 countries, with substantial gaps in systematic health education delivery [24]. A 2025 survey across Europe found that over 50% of respondents still consider menstruation a taboo topic, with more than 5,000 euphemisms documented to avoid naming menstruation directly [25]. In Ukraine, 93% of adolescent girls expressed a need for more information about menstruation, with 38% relying on the internet as their primary source due to a lack of guidance from parents and teachers [26]. In Kyrgyzstan, menstruation remains deeply taboo, with adolescent girls entering puberty without understanding what is happening to their bodies; many do not attend school during their periods, significantly affecting educational performance [27]. Recent data from UNICEF’s 2024 report also reveals that many schools still lack basic hygiene coverage in Central Asia and require safer menstrual management, including private bins, soap, and water within toilet stalls [15].

Georgia, situated within this regional context and classified as an upper-middle-income country by the World Bank [28], reflects many of these broader patterns while also exhibiting distinct national characteristics. Since gaining independence from the Soviet Union in 1991, the country has undergone substantial health system reforms; however, reproductive health education in schools remains limited. Within the predominantly Georgian Orthodox social context, discussions of reproductive and menstrual health are often avoided, inadvertently shifting adolescent education to peers and media sources [29]. Reports from online media further indicate that cultural taboos persist, particularly in rural areas, where conversations about reproductive health are frequently considered shameful. As a result, many young women lack access to accurate information and appropriate healthcare services for menstrual health concerns [30]. Against this backdrop, systematic menstrual health education programmes are absent from most Georgian schools, and published research on menstrual health experiences among adolescent girls in Georgia remains notably scarce in the scientific literature [31].

Guided by the Social Ecological Model (SEM) [32], this qualitative study explores menstrual health knowledge, attitudes, and experiences among adolescent girls in Georgian public schools and examines the role of schools and teachers in shaping these experiences. The SEM is a multilevel framework positing that individual health behaviors and outcomes are shaped by interacting factors across five nested levels – individual, interpersonal, organizational, community, and policy – and is widely used in public health research to capture how personal, social, and structural determinants jointly influence health [32]. Specifically, the study aims to document girls’ knowledge and perceptions of menstruation; identify school-based menstrual health challenges and unmet needs; assess the adequacy of school infrastructure and resources for menstrual health management; and examine teachers’ experiences, attitudes, and preparedness regarding student menstrual health.

## Methods

### Study design and setting

This qualitative study employed focus group discussions (FGDs) and individual in-depth interviews (IDIs) to explore menstrual health knowledge, attitudes, and experiences among adolescent girls and teachers in Georgian public schools. A descriptive qualitative design was selected for its ability to capture the nuanced, contextual, and experiential dimensions of menstrual health. These areas are often inadequately addressed through quantitative methods alone [33].

This study was conducted in public schools in three cities and their surrounding rural areas in Georgia: Tbilisi (village: Tserovani), Telavi (village: Kondoli), and Batumi (villages: Khala, Uchkhiti, Khelvachauri). These locations were purposively selected to represent Georgia’s geographic and socioeconomic diversity. Tbilisi, the capital city, is the largest urban center, serving as the country’s political, economic, and cultural hub. Telavi, located in the Kakheti region of eastern Georgia, is a smaller city serving a primarily agricultural area. Batumi, situated on the Black Sea coast in the Adjara region, is Georgia’s second-largest city and a major tourist destination. The inclusion of surrounding rural villages allowed for examination of potential differences in menstrual health experiences between urban and rural settings. Fieldwork took place from March to October 2020. This period coincided with the early phase of the COVID-19 pandemic in Georgia; however, school closures did not affect participant recruitment or data collection for this study.

### Sampling and recruitment

We employed non-probability sampling methods, specifically purposive and snowball sampling, to recruit study participants. Initially, we used Facebook and focal points from previous projects in public schools to disseminate information and identify eligible individuals. NGO Youth for Public Health organized the recruitment process by contacting focal points. However, we encountered challenges due to menstrual stigma and taboo. Despite initial interest from some schoolgirls, several later declined to participate due to shyness about discussing this sensitive topic. To address this, we implemented snowball sampling, asking participants to invite their peers, in addition to further purposive recruitment.

Our study participants included schoolgirls and teachers from public schools in the specified geographical locations. Inclusion criteria for schoolgirls were: (1) aged ≥ 14 years; (2) currently enrolled in a public school in one of the study areas; (3) post-menarche (having begun menstruation); and (4) able to provide informed assent with parental/guardian consent. The minimum age of 14 as an inclusion criterion was established based on local legislation, which assigns this age as the threshold for providing informed consent. Inclusion criteria for teachers were: (1) currently employed in a public school in one of the study areas; (2) teaching biology, civic education, or being a homeroom teacher to students in grades 7–12; and (3) willing to participate.

At the end of in-depth interviews and focus-group discussions, participants received a handbook on menstruation developed by the researchers to provide essential knowledge about menstruation to schoolgirls. This handbook included evidence-based information on menstrual physiology, hygiene practices, common menstrual disorders, and available resources. The provision of this educational material served as both a form of reciprocity for participation and an intervention to address knowledge gaps identified during data collection.

### Data collection and procedures

Before participation, each participant received information about the study goals, research qualifications, and their rights. This included a detailed explanation of the research purpose, procedures, the voluntary nature of participation, the right to withdraw at any time without consequence, confidentiality protections, and how the data would be used. For participants under age 18, written informed consent was obtained from a parent or legal guardian, and written assent was obtained from the adolescent participant. For participants aged 18 and above, written informed consent was obtained directly from the participant.

Two female researchers (EI, EK) collected all the data. One data collector held an advanced degree in public health, and another in human rights. Both had extensive experience in qualitative research methods and research ethics. Female researchers were specifically selected for data collection, given the sensitive, gendered nature of menstruation topics, and to facilitate comfort and openness among participants [34]. In response to the COVID-19 pandemic, data collection procedures were adapted to align with national public health guidance: researchers and participants wore face masks and maintained physical distancing during all in-depth interviews and focus group discussions.

Individual interviews were used for all teacher participants (*n* = 9) and for a subset of schoolgirls (*n* = 11) who preferred one-on-one discussions. Focus group discussions (*n* = 6) were co-facilitated by both researchers, and in-depth interviews were conducted individually. Focus groups included 5–8 schoolgirl participants grouped by similar age and geographic area to encourage peer interaction and discussion.

The duration of in-depth interviews ranged from 55 to 90 min; focus group discussions lasted between 90 and 120 min. Voice recorders were utilized during both in-depth interviews and focus group discussions. Participants were informed about audio recording before providing consent and were given the option to decline recording (none declined). Audio files were stored on password-protected devices. Identifying information was removed during transcription, and audio files were permanently deleted following verification of transcript accuracy.

### Data collection instruments

We developed three distinct data collection instruments tailored for (1) focus group discussions; (2) in-depth interviews with schoolgirls; and (3) in-depth interviews with teachers. All instruments incorporated open-ended questions designed to assess menstrual stigma, including experiences of shame, bullying, knowledge about menstruation and menstrual products; the impact of public school infrastructure on schoolgirls’ menstrual hygiene management practices; and schoolgirls’ and teachers’ attitudes and beliefs about menstrual health and education. Broad questions were accompanied by probes, with formulations tailored to address the subtle nuances specific to each target population (see supplementary files for complete data collection instruments). The focus group discussion guide emphasized peer interactions and shared experiences, using activities and prompts designed to facilitate group discussion. The individual interview guides for schoolgirls allowed for deeper exploration of personal experiences that participants might be reluctant to share in group settings. The teacher interview guide focused on pedagogical approaches, institutional barriers, and teachers’ own preparedness and comfort levels with menstrual health topics.

### Data analysis

Audio recordings of each in-depth interview and focus group discussion were transcribed verbatim in Georgian by researchers (EI, EK). Data analysis employed a thematic coding method using a hybrid inductive-deductive approach [35, 36]. This approach combined deductive analysis guided by pre-existing themes used in data collection instruments, with inductive analysis that allowed new themes to emerge from the data itself [37]. The hybrid approach has been widely used in public health qualitative research examining complex health behaviors in social contexts [38, 39] and was first systematically described by Fereday and Muir-Cochrane (2006) [36] and further developed by Swain (2018) [35].

To report study findings, we used the SEM [32] as a deductive analytical framework, structuring the codebook around five levels: individual (knowledge, attitudes, experiences), interpersonal (interactions with peers, teachers, family), organizational (school policies, infrastructure, curriculum), community (cultural norms, beliefs, taboos), and policy (national and local education policies, health policies).

The analytical process proceeded as follows: (1) familiarization of all transcripts by both researchers; (2) development of an initial codebook, including main topics from data collection instruments as themes; (3) independent coding in an Excel document; (4) code checking and discussing together during biweekly meetings. These meetings served multiple purposes: (a) reviewing coding decisions for consistency; (b) discussing emergent codes and determining whether to add them to the codebook; (c) refining code definitions; (d) identifying areas of disagreement and reaching consensus; and (e) discussing patterns and preliminary themes. Once coding was complete, the codes were grouped into broader themes and sub-themes.

### Ethical considerations

Study participation was voluntary. Written informed consent was obtained from all participants before data collection (additional consent from the minors’ parent or legal guardian). The Institutional Review Board (IRB) at the National Center for Disease Control and Prevention in Georgia approved the protocol (IRB #2020-006).

## Results

### Participants characteristics

The final study sample included 39 schoolgirls aged 14–18 years, all of whom had begun menstruating. Table 1 shows the number and distribution of schoolgirl participants across three geographic areas. Data were collected through six focus group discussions and 11 individual in-depth interviews with schoolgirls.

**Table 1:** Number of participants in in-depth interviews and focus group discussions by geographic region

Exploring menstrual health knowledge, attitude, and experiences among adolescent girls in public schools in Georgia: A qualitative study (2020).				
**Region**,** City**,** Village**	**Teachers** (*n* = 9)	**Students** (*n* = 39)		**Total**
	**In-depth Interview**	**In-depth Interview**	**Focus group discussion**	
Adjara	3	3	10	16
Batumi	2	3	6	11
Villages: Khala, Uchkhiti, Khelvachauri	1	0	4	5
Tbilisi	3	5	9	17
Tbilisi	2	4	5	11
Village: Tserovani	1	1	4	6
Kakheti	3	3	9	15
Telavi	2	2	4	8
Village: Kondoli	1	1	5	7
Total	9	11	28	48

Nine female teachers aged 25–56 years participated in individual in-depth interviews. Teaching experience ranged from 3 to 32 years. Five teachers taught civil education, one taught biology, and the remaining three were homeroom teachers, of whom two also taught Georgian literature, and one also taught mathematics (Table 1).

Analysis of interview and focus group data identified themes organized according to the Social Ecological Model across five levels: individual, interpersonal, organizational, community, and policy. Findings are presented first from schoolgirls, then from teachers within each theme. Main themes with extended participant quotes are shown in Table 2.

**Table 2:** Participant quotes. Exploring menstrual health knowledge, attitude, and experiences among adolescent girls in public schools in Georgia: A qualitative study (2020)

Participant Type	Extended Quote
Individual Level	
Knowledge gaps and misconceptions	
Schoolgirl (Kakheti)	“A body cleansing process from harmful substances, such as toxins and bacteria, and dirty blood comes out from our bodies. This is what we were told by our mothers and grandmothers. I think this is why we bleed every month - to get rid of these toxic things. I’m not completely sure this is correct, but it makes sense because if you have bad blood, it needs to come out somehow.”
Teacher (Tbilisi)	“As for menstruation itself, I don’t know how competent I am or how much I actually know. I mean, once, when I was little, I went to a gynecologist because it was hurting a lot, and at that time they explained to me that some excess things are released from the body, which is normal, that every woman should have this… I don’t remember exactly what they told me - something about getting rid of excess substances or something like that, so that a woman’s body clears itself once a month. I don’t know exactly, I mean, not in depth.”
Interpersonal Level	
Communication barriers	
Schoolgirl (Adjara)	“It is very awkward and uncomfortable; I cannot tell him that I want menstrual products. When I need them, I have to wait until my mother comes home, or I try to get money without explaining what it’s for. Sometimes I go without products when I can’t access them, which makes going to school very difficult.”
Teacher (Telavi)	“Mothers find it even harder to talk to their children about this. Some mental change is needed for all of this, and then maybe it can be provided too. For me, my mother didn’t tell me - my grandmother told me. I think they should have information, but at what age, I don’t know. I need help with this. It’s not bad that they [boys] know, because they will have wives and children.”
Experiences of bullying and peer interactions	
Schoolgirl (Tbilisi)	“A boy noticed a classmate had stained her chair with menstrual blood. He whispered about it to a friend, who told it to another, and so on until it became a subject of widespread mockery and verbal abuse. This bullying continued for about a week. The girl was so traumatized that she didn’t return to school during that time. Even after she came back, other students would whisper and make comments. It completely changed how she behaved at school - she became very quiet and withdrawn.”
Teachers’ responses	
Teacher	”A terrible fact happened last year. I had an open lesson with 7th-grade students. A colleague kept pointing at one of my students, a girl with special educational needs, trying to signal something to me. I didn’t understand, so I called the child to the board. At that moment, she was bleeding; she had her period. I didn’t realise. How would I have thought that? After that, the lesson almost fell apart. The director told everyone to disperse and asked the girl to stay. You know how she sat? She had no reaction to anything. She was completely confused. Both parents were at work. We wrapped her in a teacher’s jacket and took her home. Everyone found out. For quite a long time after that, she didn’t come to school because she was ashamed. When she finally came back, the homeroom teacher spoke to the whole class and said there was nothing left to hide. But one boy, ironically, still asked her, “Why were you absent?”
Organizational Level	
Pedagogical practices in menstrual health education	
Schoolgirl (Batumi)	“Our teacher skipped this chapter on menstruation entirely. I resisted it and insisted we should learn it, so I read it myself. I struggled to understand the material alone and realized I would have benefited greatly from the teacher’s explanations and being able to ask questions, but they refused to teach it, saying we should learn it on our own. By the time this lesson was supposed to happen in 9th grade, most of us had already been menstruating for years without proper information.”
Teacher (Adjara)	“Teachers need effective strategies to address the shame and discomfort surrounding menstruation and facilitate open conversations. The Ministry of Education and schools should provide training and resources to equip teachers with the necessary skills to navigate these discussions confidently. Currently, we receive no guidance on how to approach this topic, what language to use, or how to handle questions that arise. We’re left to figure it out on our own, which often means we avoid the topic altogether or handle it poorly.”
Physical infrastructure and sanitation facilities	
Schoolgirl (Tbilisi)	“The toilet stalls don’t offer any privacy; anyone can see you. We are used to the chaos. Before they fixed the lock on one stall, previously none of the stalls locked; a classmate followed us in, and those waiting outside had to warn others that it was occupied. There’s often no soap, the sinks don’t work properly, and there are no bins inside the stalls to dispose of used pads. During breaks, there are long queues, so if you need to change your pad during menstruation, you have to wait a long time or skip it entirely.”
Teacher (Telavi)	”The toilet is outside the building; they should build it inside this summer. It doesn’t heat up. The toilet doors don’t lock for students, only for teachers. There are only three stalls in total, and they have no doors. There is no hand-dryer, no drying facility at all. There are bins and liquid soap, but no hot water. Toilet paper is there. In winter, the pipes freeze, and there are problems with the water supply. I myself have comfort; I go home during this time. My house is 100 m away. I probably use the school toilet once a year.”
Bathroom access policies and teacher decision-making	
Teacher (Telavi)	“We always let them go. When a person is worried, you can see it on their face, and I always let them. When they want to leave just for fun, you can see that too - they tell me and laugh. I don’t let two go together, but if they explain that the toilet door doesn’t lock and someone needs to stand guard outside, I let them both. Some colleagues shout that we are spoiling the students and refuse to let them go. But what do I know about what they are feeling at that moment? We don’t have a formal restriction on this in internal rules, but we do have a restriction on unjustified requests.”
Community Level	
Menstruation as a cultural taboo	
Schoolgirl (Kakheti)	”In our culture, menstruation is a thing that should be hidden. We don’t use the word ‘menstruation’ among ourselves - we say ‘it,’ or ‘guests,’ or ‘cycle.’ Everyone understands what we mean without saying it directly. Speaking about it openly feels wrong, as if we are breaking an unspoken rule that has always been there. Even among close friends, we lower our voices.”
Cultural beliefs about menstrual impurity and contamination	
Schoolgirl (Kakheti)	”It was believed that sacred objects become impure if touched by menstruating individuals. In our region, some believe that women are not allowed to participate in baking traditional Easter bread while menstruating. Such beliefs often prevent us from engaging in certain activities. For instance, some girls refuse to swim, as they fear they would contaminate the water and therefore harm others. Although most of us do not know the origins of these beliefs, and many do not personally believe them, we often follow these rules due to social pressure from elders and family members.”
Teacher (Telavi)	“I baked Easter bread. Yes, I baked it anyway, but I was very worried that I was in this condition [menstruating], and sort of I didn’t want to, but I baked it anyway. I always enter the church; I just try not to touch blessed objects. I had a case on an excursion—one parent bought icons and wanted to give one to me. The priest understood and said, ‘This one is not consecrated, it doesn’t matter whether you touch it or not.’ That other woman wouldn’t touch hers because she had her period. With holiness—at this time, a woman is supposedly unclean. I try not to touch certain things during this time. But I don’t actually consider that a woman is unclean. I’ve been in a temple where women are not allowed to enter, and I respect that tradition. When students asked me, ‘Should I enter or not when I’m like this?‘, I told them I would normally enter. I wouldn’t tell a child, ‘Don’t enter, you are unclean,’ would I?”
Stigma in public spaces and social settings	
Schoolgirl (Adjara)	“When there are men in a pharmacy, I hide that I want menstrual products. I cannot say ‘I’ll take menstrual pads’ in front of men. Instead, I tell them, ‘You go first, and then I will buy.’ Sometimes I’ll buy other items like chocolate or medicine just so it’s not obvious what I’m really there for. In our village, everyone knows everyone, so if people see you buying pads, they’ll know you’re menstruating, and that feels very embarrassing. The whole community will know.”
Schoolgirl (Kakheti)	“In small villages where social connections are tighter, everyone talks about everything. If something embarrassing happens to you at school, within hours, the whole village knows. This makes us extra careful about anything related to menstruation because we don’t want to become the subject of village gossip. Our reputation in the community matters a lot.”
Teachers’ perceptions of generational change in stigma	
*Teacher (Telavi)*	”You know what, I can’t see this [stigma] in the current generation anymore. Children talk to you so freely, I just can’t see it. They talk to each other very openly, too. Girls, of course. There may be some degree of it, but I think they are freer, and I probably compare everything to myself. Stigma is a lack of information… That’s why they are the way they are, because of a lack of information.”
Policy Level	
Gaps in teacher training and preparation	
*Teacher (Telavi)*	”I can’t deceive you, I really have never had a conversation about this in the educational process. Probably, I don’t consider myself competent to talk about this issue. The biology teacher may talk about it to some degree. I know for sure she talks to girls, but how much she talks to boys… Generally, she talks about these issues because the curriculum includes them, but how much she actually answers questions, I don’t know. Should schools take care of teacher education? Yes, I completely agree.”
Curriculum gaps and policy deficiencies	
*Teacher (Telavi)*	“As a civic education teacher, we do have a health topic, but unfortunately, not on this issue. We have Down syndrome, drug addiction, AIDS, but not this. But I definitely talk to my students about it, and the reason is: there are often cases when a child is absent or asks to leave the lesson. One reason is that they have pain. It is embarrassing for them to tell the teacher the reason, and they say ‘you know,’ and it’s understood. I have this thing where I send the boys out, keep the girls, and talk to them about it. Separation is necessary. The textbook doesn’t actually include this topic, but children become so open in civic education that there is no restriction or complex stopping them from asking.”

### Theme 1: individual level – knowledge and beliefs about menstruation

#### Subtheme 1.1: knowledge gaps and misconceptions

Schoolgirls reported critical gaps in menstrual health knowledge, particularly regarding menstrual physiology. Many participants believed menstrual blood is impure and contains toxic materials requiring expulsion from the body. One schoolgirl from Kakheti explained: *“A body cleansing process from harmful substances*,* such as toxins and bacteria*,* and dirty blood comes out from our bodies.”* While most schoolgirls noted the link between menstrual and reproductive health, none understood the exact role of menstruation in women’s reproductive function. Teachers also held similar misconceptions about menstrual physiology. One teacher from Telavi described menstruation as *“dirty blood coming out of the woman’s organism.”* Another teacher from Kakheti associated menstruation with youth, stating, *“It is associated with youth*,* somehow. When this goes away*,* the organism*,* I think*,* starts to get dirty”* [laughs].

### Theme 2: interpersonal level – communication and social support

#### Subtheme 2.1: communication barriers

Schoolgirls reported reluctance to discuss menstruation openly, even with close friends and family members. Difficulties were particularly pronounced with male family members. One participant whose mother worked abroad as an immigrant expressed an inability to discuss menstrual health with her father and brothers, hindering her access to necessary healthcare services. A schoolgirl from Adjara explained: *”My father and I do not have such a relationship* [to talk about menstruation]. *It is very awkward and uncomfortable; I cannot tell him that I want menstrual products.”* Another participant from Kakheti stated: *”I cannot mention it* [menstruation] *in front of my father. Once*,* I missed school*,* and my father asked for a reason; I lied to him that I was going somewhere else. I could not say that my belly hurts.”*

Teachers also stated difficulties in communication between mothers and daughters. Moreover, they reported their own discomfort discussing menstruation with male colleagues and family members. One teacher from Telavi stated, *”I have some complexes. I don’t focus attention on it even if I have physical discomfort. I never said that I have this* [menstruation].” Teachers also talked about difficulties in talking about menstruation with schoolgirls. One teacher from Tbilisi reported: *“Talking about sensitive topics like menstruation requires careful consideration and preparation. I tend to avoid potential problems by providing only surface-level information*,* but I recognize the need for a more thorough approach.”*

#### Subtheme 2.2: experiences of bullying and peer interactions

Schoolgirls reported experiences of being bullied following menstrual bleeding on clothes or chairs at school. These experiences often led them to avoid public transportation, social gatherings, school attendance, and participation in school activities during menstruation. One schoolgirl from Kakheti described: *“My friend often misses the first few days of school during her period due to heavy bleeding and the fear of staining her clothes.”* A schoolgirl from Tbilisi recounted: *”A boy noticed a classmate had stained her chair with menstrual blood. He whispered about it to a friend*,* who told it to another*,* and so on until it became a subject of widespread mockery and verbal abuse.”*

#### Subtheme 2.3: teachers’ responses

Schoolgirls reported that teachers encouraged “hiding” menstruation and blamed students for menstrual leaks. One schoolgirl from Adjara stated, *“We would get scolded by teachers if we accidentally leaked during our period. They’d say things like*,* ‘Why didn’t you organize yourself better?”*

Teachers described placing responsibility on girls to anticipate and prevent menstrual leaks through self-monitoring. One teacher from Adjara stated: *“A child is obliged to have information about her organism. The child herself*,* the mother too! We also often say that you see*,* it happened to you once*,* it happened to you twice* [spotting the clothes with menstruation]…*”*.

Teachers described prioritizing concealment and ad hoc responses to menstrual emergencies. One teacher from Telavi reported: *“I always carry a scarf or some extra clothing*,* and I help them.”* Teachers also expressed uncertainty about appropriate responses to bullying. A teacher from Tbilisi reported: “*My goal is to defuse the situation so that a child wouldn’t remain singled out.”* The teacher later reflected on her own limitations: *“It’s possible I don’t have the skills necessary… Maybe I don’t have that knowledge.”*

### Theme 3: organizational level – school environment and support systems

#### Subtheme 3.1: pedagogical practices in menstrual health education

Schoolgirls reported that teachers frequently skipped menstruation lessons or segregated students by gender. A schoolgirl from Adjara criticized this practice: *“Everyone should learn about it* [menstruation] *together. If you can’t discuss it openly with boys*,* it implies that menstruation is something shameful or taboo*.”

Teachers also acknowledged that biology teachers often skip or inadequately cover menstrual health content. One teacher from Telavi recounted: *“Biology teachers really avoid these topics. They deliver this lesson superficially.”* Most homeroom teachers reported addressing the topic of menstrual health separately: *“I have this practice where I let the boys go*,* keep the girls*,* and talk to them about this. Doing it separately is definitely necessary.”*

However, teachers expressed willingness to provide menstrual health education but acknowledged a lack of training and resources. One teacher from Adjara stated: *“Teachers need effective strategies to address the shame and discomfort surrounding menstruation and facilitate open conversations.”* Teachers also reported feeling discomfort when managing classroom behavior during these lessons.

While most schoolgirls noted the significance of delivering classes on menstrual health to both genders together, teachers expressed a lack of confidence and uncertainty about whether boys and girls should learn together. One teacher from Adjara reflected: *”I’ll tell you this too*,* that on the first stage*,* girls feel more comfortable when only they are being talked to. But I think we adults have this feeling more than children*,* even awkwardness with peer boys.”*

#### Subtheme 3.2: physical infrastructure and sanitation facilities

Schoolgirls reported that school facilities were inadequate for maintaining hygiene, particularly during menstruation. Issues included unsanitary bathroom conditions, malfunctioning faucets, frequent absence of soap, and broken or absent door locks. One schoolgirl from Tbilisi described: *“The toilet stalls don’t offer any privacy; anyone can see you. We are used to the chaos. Before they fixed the lock on one stall* [previously none of the stalls locked], *a classmate followed us in and waiting outside had to warn others that it was occupied.”* Many schoolgirls, particularly from rural areas, reported avoiding school bathrooms and preferring to go home when needing to change menstrual products. One participant from Tbilisi stated: *“I often try to avoid entering the school toilet. I always go home if it’s almost the end of the day*,* or I go to the shopping center. I never use a toilet in school.“*.

Teachers confirmed inadequate facilities. Some teachers reported choosing their school based on proximity to home, allowing them to avoid using school bathrooms by going home during breaks.

#### Subtheme 3.3: availability and accessibility of menstrual products

Schoolgirls reported that menstrual hygiene products and painkillers are often available in public schools, stored with school nurses or security officers, but only accessible on demand for unexpected needs. While the availability was viewed positively, several issues emerged. Products were often of poor quality, and in schools where the nurse or security officer was male, girls expressed discomfort requesting products due to menstrual stigma. Schoolgirls suggested making menstrual hygiene products readily accessible in bathrooms, for instance, through dispensers.

Similarly, teachers confirmed that they provide emergency access to menstrual products. One teacher from Telavi stated, ”*They are very open and frank when talking about menstrual products. Everyone knows that something* [menstrual products] *is there*,* and they get help.”* However, teachers noted challenges when male staff managed product distribution. One teacher from Kakheti questioned: *”A male nurse/pharmacist would be a barrier for students. But maybe it wouldn’t. The girls are a bit more cheerful. But not everyone is like that. They will have it* [barrier].*”*

#### Subtheme 3.5: bathroom access policies and teacher decision-making

Teachers described school policies regarding bathroom access during class, revealing how they navigate institutional rules. One teacher from Telavi explained: *”We always allow them. When a person is worried*,* you can see it on their face*,* too*,* and I always allow it.”* A teacher from Adjara described stricter requirements: *“I need to know the reason why they’re leaving class. We don’t have the right to dismiss students. We need to know the reason.”* Another teacher from Telavi reported liberal bathroom access: *“Yes. I allow it. Some teachers think that they’re spoiling them and don’t allow them.”*

### Theme 4: community level – cultural norms, taboos, and stigma

#### Subtheme 4.1: menstruation as a cultural taboo

Schoolgirls reported that menstruation is considered a taboo topic in Georgian culture – ”a thing that should be hidden.” Participants avoided using the word “menstruation,” replacing it with euphemisms such as ”it,” ”guests,” or “cycle.” This cultural taboo was described as deeply rooted and associated with collective notions of shame and impropriety.

#### Subtheme 4.2: cultural beliefs about menstrual impurity and contamination

Schoolgirls reported widespread cultural beliefs that menstruating individuals contaminate sacred objects or spaces. These beliefs were described as community-held traditions rather than personal convictions. Critically, most participants reported that although they did not know the origins of these beliefs and many did not personally endorse them, they followed these restrictions due to social pressure from family and community members. One schoolgirl from Kakheti explained: *”A body cleansing process from harmful substances*,* such as toxins and bacteria*,* and dirty blood comes out from our bodies”* - demonstrating how community beliefs become internalized even when not personally believed. Teachers reported that cultural beliefs about menstrual impurity extended to religious practices. One teacher from Telavi described her own practices: *“I baked Easter bread. Yes*,* I baked it anyway*,* but I was very worried that I was in this condition* [menstruation], *and as if I didn’t want to*,* but I baked it anyway.”*

#### Subtheme 4.3: stigma in public spaces and social settings

Schoolgirls reported feeling uncomfortable purchasing menstrual products when men were present in pharmacies. In small villages where social connections are tighter, this discomfort was amplified due to fear of community knowledge and judgment. This led some schoolgirls to purchase unrelated items (e.g., chocolate) to avoid drawing attention to their needs. *One schoolgirl from Adjara explained: “When there are men in a pharmacy*,* I hide that I want it* [menstrual products]. *I cannot say it* [I’ll take menstrual products] *in front of men. Instead*, *I tell them*,* “You go first*,* and then I will buy.”*

#### Subtheme 4.4: teachers’ perceptions of generational change in stigma

Teachers offered mixed perspectives on whether menstrual stigma persists in the current generation. One teacher from Telavi observed: *”You know what? I can’t see this* [shame, stigma] *in this generation. Children talk to you so freely*,* I can’t see it. They talk to each other very openly*,* too. Girls*,* of course. There might be some dose of it*,* but I think they are freer*,* and I probably compare everything to my own self.”*

However, another teacher from Kakheti reported observing stigma among students: *”I think stigma exists. I’ve felt that children are restricted and evasive. I’ve often had conversations with students about this. I notice that current girls have breasts and are physically more mature*,* and often sit hunched over. I tell them to dress appropriately and dress accordingly because there are boys there too*,* and awkwardness exists.”* Teachers attributed stigma to a lack of awareness. One from Tbilisi stated: *“Stigma is a lack of information… That’s why they are this way*,* because of a lack of information.”*

### Theme 5: policy level – educational system and teaching training

#### Subthemes 5.1: gaps in teacher training and preparation

Teachers explicitly stated they lack competence and training. One teacher from Telavi admitted, *”I can’t deceive you*,* I really haven’t had a conversation about this in the educational process. You know what*,* probably I don’t consider myself competent to talk about this issue.”* Another from Telavi acknowledged: “*It’s possible my skills weren’t sufficient to provide information. I don’t know. Maybe I didn’t have that knowledge*,* and that’s why.”*

Teachers emphasized that the Ministry of Education should provide training and resources to equip educators with the necessary skills. One teacher from Adjara stated: *“The Ministry of Education and schools should provide training and resources to equip teachers with the necessary skills to navigate these discussions confidently.”*

#### Subtheme 5.2: curriculum gaps and policy deficiencies

Schoolgirls reported that menstrual health lessons were delivered too late, typically in 9th grade, when most had already begun menstruating. One participant from Tbilisi noted: *“Menstruation is taught in school around 9th grade when most girls have already started their periods*,* although the information provided is minimal.”* Schoolgirls and teachers reported that menstrual health education is minimally addressed in the formal curriculum, occurs too late in students’ development, and lacks standardized, comprehensive content. This reflects policy-level gaps in educational planning and curriculum development. Teachers reported that curriculum constraints prevented comprehensive menstrual health education. One teacher from Telavi noted: *”Our curriculum doesn’t include this.”* A teacher from Kakheti, who teaches civic education, noted: *“As a civic education teacher*,* we also have this topic - health*,* but unfortunately not on this issue* [menstrual health]. *We have topics about Down syndrome*,* drug addiction*,* and HIV*,* but not on this issue.”*

## Discussion

This qualitative study examined menstrual health knowledge, attitudes, and experiences among schoolgirls and teachers in Georgian public schools, using the SEM to understand factors across five levels: individual, interpersonal, organizational, community, and policy. Our findings suggest gaps in menstrual health knowledge and widespread misconceptions among both schoolgirls and teachers in Georgia. These findings can be understood within the broader EECA regional context, where the Soviet Union’s systematic suppression of reproductive and sexual health education in most countries created enduring barriers to menstrual health literacy and open communication that persist across former Soviet republics, including Georgia [23].

### Individual level – knowledge gaps and misconceptions

At the individual level, our findings suggest significant knowledge gaps and misconceptions about menstrual physiology among schoolgirls and teachers alike. These misconceptions reflect not only insufficient formal education but also the intergenerational transmission of misinformation - a pattern documented across the EECA region where Soviet-era educational policies systematically excluded reproductive health content from school curricula [23]. Georgia’s situation is further complicated by a strong Orthodox context that views menstruation as impure. Holistic menstrual health education can significantly improve knowledge, reduce stigma, and enhance positive attitudes toward menstruation among adolescent girls [40–42], particularly in traditional contexts where sexual education and broader discussions of sexuality face strong resistance.

### Interpersonal level – communication barriers and social support

Communication barriers and silence surrounding menstruation reflect broader gender-based communication patterns documented across Eastern Europe, where menstruation remains a topic relegated to female-only spaces [43]. The bullying experiences reported by participants, including sustained mockery and verbal abuse following menstrual leaks, represent serious violations of girls’ dignity with direct educational consequences [44, 45]. These experiences illuminate the intersection of menstrual stigma with gender-based school violence, an issue that has received insufficient attention in educational policy across the region [7].

Teachers’ focus on helping girls conceal menstruation shifts responsibility onto students and reinforces stigma, rather than addressing systemic gaps in menstrual health education and school infrastructure. Educational approaches that aim to reduce stigma instead emphasize critical reflection, empowerment, and supportive teaching practices, helping students navigate and challenge discriminatory social norms rather than simply hiding them [46].

### Organizational level – school systems and institutional practices

At the organizational level, our findings illustrated how school-based practices and infrastructure both reflect and perpetuate menstrual stigma. The documented pedagogical practices create systematic barriers to comprehensive menstrual health education. These practices occur despite menstrual health content being included in the curriculum, suggesting that informal institutional norms override formal educational requirements.

The inadequacy of school physical infrastructure represents a critical organizational failure with direct health and educational implications. These conditions align with broader regional findings: while UNICEF reports 84% basic hygiene coverage in Central and Southern Asia, most facilities lack essential menstrual hygiene management services such as private disposal bins, soap, and water in stalls [26, 27, 47]. As many girls in our study avoided school bathrooms entirely, preferring to go home or to commercial establishments, which for some meant limiting fluid intake or missing class time.

These organizational-level findings have clear practice implications: schools require comprehensive menstrual health management policies that address not only product provision but also infrastructure upgrades, flexible bathroom access, privacy protections, and the integration of menstrual health into standard school health protocols. School administrators and education officials must recognize menstrual health management as essential educational infrastructure, not an optional add-on.

### Community level – cultural norms, taboos, and stigma

At the community level, menstruation remains a deeply entrenched cultural taboo in Georgia. Regional data from Eastern Europe show that in some contexts, over 50% of people consider menstruation a taboo topic, with researchers documenting more than 5,000 euphemisms to avoid direct discussion. This linguistic avoidance both reflects and reinforces stigma. Many individuals follow menstrual restrictions not out of personal conviction but due to social pressure and fear of judgment, highlighting how cultural norms are enforced socially rather than universally internalized. Stigma also extends into public spaces, limiting girls’ and women’s ability to access health products with dignity.

### Policy level – educational system and teacher preparation

At the policy level, our findings reveal systemic failures in menstrual health education policy and teacher preparation. The study showed a critical policy failure: teachers are expected to deliver menstrual health education without any formal training or professional development in this area. Moreover, the curriculum itself contains significant gaps, with menstrual health education delivered too late (typically 9th grade, after most girls have begun menstruating), too briefly, and with insufficient depth. Even subjects that nominally cover related content, such as civic education classes addressing health, exclude menstrual health in favor of topics like drug use disorders and HIV. We suggest that this curriculum structure may inadvertently signal, to both teachers and students, a lower institutional priority for menstrual health relative to other topics covered in the curriculum.

Given this context, our findings point to several potential policy priorities. These include the development of comprehensive menstrual health education standards, integration of menstrual health content throughout the curriculum (beginning in primary school), mandatory teacher training and continuing professional development, and mechanisms to support implementation. Beyond education policy, our findings suggest a need for attention to menstrual product affordability and accessibility, school infrastructure standards, and the cultural norms that perpetuate stigma.

### Strengths and limitations

Our study has several important strengths. The use of the Social Ecological Model provides a comprehensive framework for understanding how factors at multiple levels interact to shape menstrual health experiences. Including both schoolgirls and teachers as participants allowed us to examine the issue from multiple perspectives within the educational system. The qualitative approach enabled deep exploration of sensitive topics and uncovered the mechanisms through which stigma operates at interpersonal, organizational, community, and policy levels.

However, our study also has limitations that should be considered when interpreting findings. First, due to menstrual stigma and shame, many potential participants declined to join the study. This may mean our findings underestimate the severity of knowledge gaps and experiences of stigma among the broader population of Georgian schoolgirls, as those most affected by stigma may have been least likely to participate. Second, our sample did not include individuals with disabilities, ethnic minorities, or transgender or gender non-conforming individuals, all of whom may face unique and compounded challenges related to menstruation that remained unexplored in the Georgian context. Third, despite using probing and indirect approaches, the sensitive nature of menstruation may have limited participants’ willingness to fully disclose their experiences, particularly around cultural beliefs and personal practices; social desirability may also have shaped responses. Fourth, our purposive and snowball sampling strategy, while well-suited to reaching a stigmatized and hard-to-engage population, limits the transferability of findings: participants were drawn from three regions and their surrounding rural areas, and findings should not be interpreted as representative of all schoolgirls, teachers, or schools across Georgia.

## Conclusion

This qualitative study suggests that, among schoolgirls and teachers who participated, menstrual health challenges operate across multiple ecological levels, from individual knowledge gaps and interpersonal communication barriers to organizational infrastructure inadequacies, community-level cultural taboos, and systemic policy gaps. Within the schools and communities studied, these multilevel barriers appeared to undermine schoolgirls’ menstrual health, educational participation, and overall well-being. Addressing these challenges is likely to require coordinated policy action: Ministry of Education efforts to develop comprehensive menstrual health education standards and teacher training; school-level investment in dignified infrastructure and menstrual health management policies; and community-engaged initiatives to challenge menstrual taboos. Our findings suggest that gender equity efforts in Georgia would benefit from greater attention to menstrual health as a fundamental aspect of bodily autonomy, educational access, and human dignity. Multilevel interventions of this kind may help move Georgian schools toward environments where girls can manage menstruation with dignity and participate fully in education with fewer stigma-related barriers.

## Supplementary Information

Supplementary Material 1.

## Data Availability

The data supporting our study’s findings are available from the youth organization Youth for Public Health. The data were collected specifically for this study and securely stored. Due to data privacy concerns and the confidentiality of the information, the interview records have been deleted after analysis. However, the data analysis code and documentation are available upon reasonable request from the corresponding author.

## References

[1] Hennegan J, Winkler IT, Bobel C, et al. Menstrual health: a definition for policy, practice, and research. Sex Reproductive Health Matters. 2021;29:31–8. 10.1080/26410397.2021.1911618 PMC8098749 33910492

[2] Babbar K, Martin J, Ruiz J, et al. Menstrual health is a public health and human rights issue. Lancet Public Health. 2022;7:e10–1. 10.1016/S2468-2667(21)00212-7 PMC8552814 34717798

[3] World Bank. Menstrual Hygiene Management Enables Women and Girls to Reach Their Full Potential. World Bank. https://www.worldbank.org/en/news/feature/2018/05/25/menstrual-hygiene-management. Accessed 4 February 2026.

[4] Van Eijk AM, Zulaika G, Lenchner M, et al. Menstrual cup use, leakage, acceptability, safety, and availability: a systematic review and meta-analysis. Lancet Public Health. 2019;4:e376–93. 10.1016/S2468-2667(19)30111-2 PMC6669309 31324419

[5] Shah V, Nabwera H, Sonko B, et al. Effects of Menstrual Health and Hygiene on School Absenteeism and Drop-Out among Adolescent Girls in Rural Gambia. IJERPH. 2022;19:3337. 10.3390/ijerph19063337 PMC8954348 35329020

[6] Miiro G, Rutakumwa R, Nakiyingi-Miiro J, et al. Menstrual health and school absenteeism among adolescent girls in Uganda (MENISCUS): a feasibility study. BMC Women’s Health. 2018;18:4. 10.1186/s12905-017-0502-z PMC5753466 29298699

[7] UNESCO. Puberty education and menstrual hygiene management. 2014.

[8] WaterAid U, Domestos, Water Supply and Sanitation Collaborative Council (WSSCC). We can’t wait: A report on sanitation and hygiene for women and girls. Advocacy Report, London: WaterAid; 2013. https://washmatters.wateraid.org/sites/g/files/jkxoof256/files/we%20cant%20wait.pdf. Accessed 4 February 2026.

[9] Plan International UK. Nearly two million girls in the UK miss school because of their period. 2021. https://plan-uk.org/press/two-million-girls-in-the-uk-miss-school-because-of-their-period. Accessed 28 January 2026.

[10] Sommer M, Torondel B, Hennegan J, et al. How addressing menstrual health and hygiene may enable progress across the Sustainable Development Goals. Global Health Action. 2021;14:1920315. 10.1080/16549716.2021.1920315 PMC8253211 34190034

[11] Langford R, Bonell C, Jones H, et al. The World Health Organization’s Health Promoting Schools framework: a Cochrane systematic review and meta-analysis. BMC Public Health. 2015;15:130. 10.1186/s12889-015-1360-y PMC4339015 25886385

[12] Huseth-Zosel AL, Secor‐Turner M. Teacher Perceptions of and Experiences with Student Menstruation in the School Setting. J Sch Health. 2022;92:194–204. 10.1111/josh.13120 34806189

[13] Mahon T, Fernandes M. Menstrual hygiene in South Asia: a neglected issue for WASH (water, sanitation and hygiene) programmes. Gend Dev. 2010;18:99–113.

[14] Chandra-Mouli V, Patel SV. Mapping the knowledge and understanding of menarche, menstrual hygiene and menstrual health among adolescent girls in low- and middle-income countries. Reprod Health. 2017;14:30. 10.1186/s12978-017-0293-6 PMC5333382 28249610

[15] World Health Organization (WHO), United Nations Children’s Fund (UNICEF). Progress on household drinking water, sanitation and hygiene 2000–2024: special focus on inequalities, Geneva. 2025. https://cdn.who.int/media/docs/default-source/wash-documents/wash-coverage/jmp/jmp-2025-wash-households-lowres-launch.pdf?sfvrsn=12ccab42_3&download=true. Accessed 4 February 2026.

[16] Mohammed S, Larsen-Reindorf RE, Awal I. Menstrual hygiene management and school absenteeism among adolescents in Ghana: results from a school-based cross-sectional study in a rural community. Int J Reprod Med. 2020;2020:1–9. 10.1155/2020/6872491 PMC7204135 32411782

[17] Adane Y, Ambelu A, Azage Yenesew M, et al. Menstrual hygiene management practices among schoolgirls in resource-limited settings of Bahir Dar City administration, Northwestern Ethiopia. Womens Health (Lond Engl). 2024;20:17455057241308343. 10.1177/17455057241308343 PMC11653455 39691973

[18] Bobel C. *The Managed Body: Developing Girls and Menstrual Health in the Global South*. 1st ed. 2019. Cham: Springer International Publishing: Imprint: Palgrave Macmillan, 2019. Epub ahead of print; 2019. 10.1007/978-3-319-89414-0.

[19] Mason L, Nyothach E, Alexander K, et al. We Keep It Secret So No One Should Know’ – A Qualitative Study to Explore Young Schoolgirls Attitudes and Experiences with Menstruation in Rural Western Kenya. PLoS ONE. 2013;8:e79132. 10.1371/journal.pone.0079132 PMC3828248 24244435

[20] Sharma S, Mehra D, Brusselaers N, et al. Menstrual Hygiene Preparedness Among Schools in India: A Systematic Review and Meta-Analysis of System-and Policy-Level Actions. IJERPH. 2020;17:647. 10.3390/ijerph17020647 PMC7013590 31963862

[21] Johnston-Robledo I, Chrisler JC. The Menstrual Mark: Menstruation as Social Stigma. In: Bobel C, Winkler IT, Fahs B editors *The Palgrave Handbook of Critical Menstruation Studies*. Singapore: Springer Singapore, pp. 181–99.

[22] Hennegan J, Shannon AK, Rubli J, et al. Women’s and girls’ experiences of menstruation in low- and middle-income countries: A systematic review and qualitative metasynthesis. PLoS Med. 2019;16:e1002803. 10.1371/journal.pmed.1002803 PMC6521998 31095568

[23] Hearne S. Broadcasting Communist Morality: Sex Education in Soviet Latvia. In: Shaw C, Toropova A, editors. Technologies of Mind and Body in the Soviet Union and the Eastern Bloc. London (UK): Bloomsbury Academic; 2023. http://www.ncbi.nlm.nih.gov/books/NBK601391/. accessed 4 February 2026). 38446941

[24] Bundeszentrale für gesundheitliche Aufklärung (BZgA). International Planned Parenthood Federation – European Network (IPPF EN). Sexuality education in Europe and Central Asia: State of the art and recent developments — Comprehensive report. Cologne, Germany: Federal Centre for Health Education (BZgA); 2018. https://whocc.bioeg.de/fileadmin/user_upload/Dokumente/BZgA_ComprehensiveCountryReport_EN.pdf.

[25] MenstrualMattersEU. Menstrual Matters EU. Our study. *MenstrualMattersEU*; 2025. https://menstrualmatters.eu/en/#study. Accessed 4 February 2026.

[26] UNICEF Ukraine. Speaking freely about menstruation. UNICEF Ukraine; 2024. https://www.unicef.org/ukraine/en/speaking-freely-about-menstruation.

[27] UNICEF Kyrgyzstan. In Kyrgyzstan, girls lift the shroud of shame on menstruation. UNICEF Kyrgyzstan; 2017. https://www.unicef.org/stories/kyrgyzstan-girls-lift-shroud-shame-menstruation. Accessed 4 February 2026.

[28] World Bank. World Bank Country and Lending Groups. World Bank Data Help Desk; 2026. https://datahelpdesk.worldbank.org/knowledgebase/articles/906519. Accessed 4 February 2026.

[29] Abzianidze T, Butsashvili M, Kajaia M, et al. Generational differences in current sexual behavior among Georgian reproductive-aged women. IJWH. 2019;11:301–8. 10.2147/IJWH.S197473 PMC6512778 31191038

[30] Gvasalia T. Sexual education in Georgia: who is against having it included in the school curricula. JAMnews; 2019. https://jam-news.net/sexual-education-in-georgia-who-is-against-having-it-included-in-the-school-curricula/. Accessed 4 February 2026.

[31] Kamushadze A. Project works to promote sexual health education in Georgia. *The Messenger*; 2008. https://www.messenger.com.ge/issues/1551_february_21_2008/1551_sex_ed.html?utm_source=chatgpt.com. Accessed 4 February 2026.

[32] McLeroy KR, Bibeau D, Steckler A, et al. An Ecological Perspective on Health Promotion Programs. Health Educ Q. 1988;15:351–77. 10.1177/109019818801500401 3068205

[33] Creswell JW, Plano Clark VL. Designing and conducting mixed methods research. Third Edition. Los Angeles: SAGE; 2018.

[34] Braun V, Clarke V. *Successful qualitative research: a practical guide for beginners*. First published. Los Angeles London New Delhi: SAGE; 2013.

[35] Swain J. A Hybrid Approach to Thematic Analysis in Qualitative Research: Using a Practical Example. 1 Oliver’s Yard, 55 City Road, London EC1Y 1SP United Kingdom: SAGE Publications Ltd. Epub ahead of print 2018. 10.4135/9781526435477.

[36] Fereday J, Muir-Cochrane E. Demonstrating Rigor Using Thematic Analysis: A Hybrid Approach of Inductive and Deductive Coding and Theme Development. Int J Qualitative Methods. 2006;5:80–92.

[37] Braun V, Clarke V. Using thematic analysis in psychology. Qualitative Res Psychol. 2006;3:77–101.

[38] Smith J, Firth J. Qualitative data analysis: the framework approach. Nurse Res. 2011;18:52–62. 10.7748/nr2011.01.18.2.52.c8284 21319484

[39] Gale NK, Heath G, Cameron E, et al. Using the framework method for the analysis of qualitative data in multi-disciplinary health research. BMC Med Res Methodol. 2013;13:117. 10.1186/1471-2288-13-117 PMC3848812 24047204

[40] Pelvic Pain Foundation of Australia. PPEP Talk Schools Program. Pelvic Pain Foundation of Australia; 2022. https://www.pelvicpain.org.au/ppep-talk-schools-program/. Accessed 5 February 2026.

[41] Roux F, Chih H, Hendriks J, et al. Mixed Method Evaluation of My Vital Cycles^®^: A Holistic School-Based Ovulatory Menstrual Health Literacy Program. IJERPH. 2023;20:5964. 10.3390/ijerph20115964 PMC10252248 37297568

[42] Roux FI, Burns SK, Hendriks JL, et al. Progressing Toward Adolescents’ Ovulatory-Menstrual Health Literacy: A Systematic Literature Review of School-Based Interventions. Women’s Reproductive Health. 2021;8:92–114.

[43] UNFPA Eastern Europe and Central Asia Regional Office. Much progress on sexual and reproductive well-being in Europe, but millions still denied rights and choices, global UNFPA report finds. UNFPA Eastern Europe and Central Asia; 2024. https://eeca.unfpa.org/en/news/much-progress-sexual-and-reproductive-well-being-europe-millions-still-denied-rights-and. Accessed 5 February 2026.

[44] Winkler IT. Introduction: Menstruation as Structural. In: Bobel C, Winkler IT, Fahs B editors *The Palgrave Handbook of Critical Menstruation Studies*. Singapore: Springer Singapore, pp. 469–73.

[45] Benshaul-Tolonen A, Zulaika G, Sommer M et al. Measuring Menstruation-Related Absenteeism Among Adolescents in Low-Income Countries. In: Bobel C, Winkler IT, Fahs B editors *The Palgrave Handbook of Critical Menstruation Studies*. Singapore: Springer Singapore, pp. 705–23.

[46] Link BG, Phelan JC. Conceptualizing Stigma. Annu Rev Sociol. 2001;27:363–85.

[47] რეკომენდაცია საჯარო. სკოლების ინფრასტრუქტურული მდგომარეობის გაუმჯობესებისა და მოსწავლეთა უსაფრთხოების დაცვისათვის საჭირო ღონისძიებათა შესახებ. https://www.ombudsman.ge/geo/rekomendatsiebi/rekomendacia-sadjaro-skolebis-infrastruqturuli-mdgomareobis-gaumdjobesebisa-da-moswavleta-usafrtxoebis-dacvisatvis-sachiro-gonisdziebata-shesaxeb. Accessed 4 August 2024.

