# Supplementary material for: Exploring menstrual health knowledge, attitude, and experiences among adolescent girls in public schools in Georgia: a qualitative study
Source: Reprod Health. 2026 Jul 25;23:190. doi: 10.1186/s12978-026-02417-7 (PMC13688914; doi:10.1186/s12978-026-02417-7)
Supplement: Supplementary file 1 [file 12978_2026_2417_MOESM1_ESM.docx]

In-depth Interview Guide for Schoolgirls

## **Demographic Information**

1. Age, grade level
2. Place of residence
3. Do you have a menstrual cycle? At what age did it start?

## **Menstruation-Related Stigma**

1. What words (adjectives and others) would you use to describe menstruation? What words come to mind when we talk about menstruation?
2. What words do you use to refer to menstruation when you talk about it?
3. With whom do you talk about menstruation (whom do you talk to when you have questions)? Where do you feel most comfortable talking about it - at school? At home? With friends? Somewhere else?
4. In your assessment, what kind of attitude exists toward menstruation in the society around you?

*Is there menstrual stigma, and how does it manifest?*

5. Do you feel free when asking questions about menstruation? Or talking about this topic?
6. In your opinion, what is shameful/what is considered shameful in relation to menstruation? Why?
7. Do you buy hygiene products yourself?

If yes:

o Do you feel embarrassed when buying hygiene products? Why?

o Does the gender of the pharmacy/supermarket salesperson matter?

If yes, why?

If no:

o What is the reason for this?

o Who buys hygiene products for you? Do you ask yourself when needed?

o If needed, would you ask your father or brother to buy hygiene products?

8. Has a teacher at school talked to you about menstruation?

*If yes, what did the teacher tell you about menstruation? What words did they use to refer to menstruation, if you remember? Did they talk to you together with boys or separately? Can you tell us about this incident?*

9. Based on your experience, what information do boys have about menstruation?
10. Do you remember any story when classmates (and others) at school mocked someone about menstruation?

If yes, can you tell us the story?

11. Have you personally felt shame related to menstruation?

If yes, can you tell us the story?

12. At school/on school trips, are you treated differently because you have your period?

In your opinion, why?

13. Do you remember a case when during class you asked the teacher to go to the restroom, they refused, and you were hesitant to say that you needed to change your hygiene product?
14. Do you remember your classmate having such a case?
15. In your opinion, why is menstruation associated with shame?

## **Impact of Stigma on Realization of the Right to Education**

16. Do you remember a case when you missed a class at school or some other educational activity due to menstruation-related shame?

If yes, tell us the story;

17. Have you heard about a case when someone you know missed some educational activity due to menstruation-related shame?

If yes, tell us the story;

18. Has anyone told you that during menstruation, it is better not to go to school and stay at home? Or conversely, you did not want to/could not go, but were still forced to attend classes?

## **Knowledge About Menstruation**

19. What is menstruation? What processes occur in the body?
20. Can you tell us about your first menstruation?

Did you have information?

If you had information, how did having this information help you? Was this information sufficient?

If you did not have information, do you think it would have helped?

21. When and where did you first learn about menstruation? Who told you (school, family, friends, internet resources, non-formal education, etc.)? Are you satisfied with the source of information, or would it have been better if the information came from someone else? If yes, from whom?
22. Can you tell us what is allowed and what is not allowed during menstruation? Which do you agree with? Which do you not agree with?
23. How should you maintain personal hygiene during menstruation (source of information)?
24. Do you think your knowledge about menstruation is sufficient?

Do you always receive competent and complete information from someone? From whom?

Do you think there are so-called "warning signs" of menstruation when a doctor's consultation is necessary? Which ones?

## **Menstrual Products**

25. What types of menstrual products do you have information about? Please list them, and from whom did you receive this information?

If the student did not list all products, we ask about each one individually.

26. Which products do you use? Which do you not use? Why?

Can you name the positive and negative aspects of each menstrual product?

When can they be used? When not?

27. At your school, is it possible to obtain menstrual hygiene products in case of emergency (e.g., from the school doctor or teacher? Does the school provide this)? Do you think it is important for something like this to exist in schools?
28. Are the menstrual products you use comfortable? Have you or your friends missed school due to discomfort?
29. Do you recall any case when someone around you did not have the financial means to purchase menstrual hygiene products?

## **School Infrastructure and Its Impact on the Realization of the Right to Education**

30. At the school where you attend, is there a restroom?
31. If yes, does the restroom door lock from the inside?
32. Does the restroom have:

Paper;

Trash bin;

Hot water;

Soap;

Toilet paper;

Functioning sewage system;

Functioning flushing system;

*Note: The researcher should ask the respondent about the components to which the answer is negative. For example, if there is no trash bin in the restroom, how does the student manage to dispose of hygiene products?*

33. (If the answer to the sub-questions of question three is negative) Do you recall a case when, because it was not possible to change in the school restroom, you stayed home during menstruation?

If yes, does this have a systematic character?

Approximately, how many activities did you miss for this reason?

In your assessment, did this affect your academic performance (grades or other assessment/encouragement)?

34. Have you heard of girls not going to school during menstruation because the school restroom is inconvenient for changing?

Focus Group Discussion Guide for Schoolgirls

## **Welcome and Introduction of Participants**

Introduction of the study's objectives and goals to participants, introduction of participants to each other, and agreement on general ground rules.

## **Discussion Introduction**

Participants share their motivation for participating in the study and reasons for their interest in the topic.

## **Main Discussion Questions**

**Stigma**

1. What associations come to mind first when you hear the word "menstruation"?
2. With whom do you talk about menstruation? With whom are you more comfortable talking about it? (friends, teachers, parents, siblings, boys)?
3. How do you understand menstrual stigma? What is it? What impact does it have on your daily life? On school attendance/academic performance?
4. Does bullying of girls happen because of menstruation? Where? Can you give an example?
5. Does the teacher allow you to go to the restroom when you ask? Do you need to provide a reason why you need to leave class?

**Knowledge About Menstruation**

6. What is menstruation? What processes occur in the body?
7. Can you tell us about your first menstruation?
8. When and where did you first learn about menstruation? Who told you (school, family, friends, internet resources, non-formal education, etc.)?
9. Can you tell us what is allowed and what is not allowed during menstruation? Which do you agree with? Which do you not agree with?
10. How should you maintain personal hygiene during menstruation (source of information)?
11. Do you think your knowledge about menstruation is sufficient?

**Menstrual Products**

12. What types of menstrual products do you have information about? Please list them, and from whom did you receive this information?
13. Which products do you use? Which do you not use? Why?
14. At your school, is it possible to obtain menstrual hygiene products in case of emergency (e.g., from the school doctor or teacher? Does the school provide this)? Do you think it is important for something like this to exist in schools?
15. Are the menstrual products you use comfortable? Have you or your friends missed school due to discomfort?
16. Do you recall any case when someone around you did not have the financial means to purchase menstrual hygiene products?
17. What can you tell us about purchasing menstrual products? How easy is it to obtain them, considering financial accessibility, shame, long distance to a pharmacy/store?

**Infrastructure**

18. At the school where you study, is there a restroom?
19. If yes, does the restroom door lock from the inside?
20. Does the restroom have:

Paper

Trash bin

Hot water

Soap

Toilet paper

Functioning sewage system

Functioning flushing system;

21. Have you heard of girls not attending school during menstruation because changing hygiene products at school is inconvenient? According to your information, how common are such cases?

In-depth Interview Guide for Teachers

## **Demographic Information**

1. Age?
2. Gender?
3. At which school are you a teacher?
4. How many years of teaching experience do you have?
5. What subject do you teach?
6. What age children do you teach?
7. Do you have a homeroom class?

## **Menstruation-Related Stigma and Its Impact**

8. What do you associate with menstruation? What comes to mind first when you hear this word?
9. Do you talk about menstruation? Where? In what context? (school, family members, friends, colleagues, etc.) What words do you use to refer to menstruation when you talk about it? When you talk to students?
10. Have you talked to students about menstruation?

If yes, with girls and boys?

If yes, do you feel free when talking to students about menstruation?

If no - why?

11. In your opinion, at what age should menstruation-related information be shared with students? Why?
12. What do you think menstrual stigma is? Does it exist? At school? In society? Can you give examples?

What causes menstrual stigma?

What negative consequences does menstrual stigma have?

13. Do you remember a case when a girl found herself in an embarrassing situation because of menstruation (meaning stained clothing, simply disclosure of information that a specific student has her period, or anything else)? If you can describe this case, what happened? What was the reaction of the rest of the class? How did you act in such a situation? Whom did you talk to and what did you advise?
14. When a female student asks to go to the restroom, do you allow her without hindrance and without questioning?

If no, why?

15. Have you heard that female students (your students, or your relatives, family members, your own experience) do not attend school during menstruation?

If yes, what do you attribute this to?

## **Knowledge/Awareness**

16. Can you tell us what menstruation is? What have you learned about it?

At what age does menstruation start for girls?

What signs appear during menstruation? Do you think that pain, prolonged bleeding, or hormonal changes that occur during menstruation can interfere with a female student's learning?

Do you think a female school student might need a gynecologist's intervention? When? For what reason?

17. Where do you get information about menstruation? Does the school or the Ministry of Education conduct special trainings on menstruation or other aspects of reproductive health? Other organizations?
18. Can you tell us what is allowed and what is not allowed during menstruation? What you consider correct, or what you do not consider correct but have heard from others?
19. How do we maintain personal hygiene during menstruation? Is there anything specific that differs from other periods of the cycle?

How often is it necessary to change hygiene products? Can this interfere with a student's class attendance? Might a student need to leave class because of this?

## **Menstrual Products**

20. Can you name menstrual products, so-called hygiene products? (Which have you heard of? Which are accessible?)

Do you think they differ from each other and what distinguishes them? When can/should each of them be used? Which is a more or less comfortable hygiene product?

21. Where can menstrual products be purchased? Is it far from the settlement? Do students have difficulty buying them?
22. Do you think hygiene products are financially accessible for students?
23. Is it possible for a student to receive menstrual products at school (e.g., from a doctor or teacher) in case of emergency?
24. Do you think the menstrual hygiene products that girls use are comfortable? Is it possible that a student might miss school due to discomfort caused by them? What can be done to avoid this?

## **School Infrastructure**

25. At the school where you teach, is there a restroom?
26. If yes, does the restroom door lock from the inside?
27. Does the restroom have:

Paper

Trash bin

Hot water

Soap

Toilet paper

Functioning sewage system

Functioning flushing system

Note: The researcher should ask the respondent whether restrooms at school are arranged separately for teachers and students. If so, answer separately for each case whether they are equipped with all the components listed above.

28. (If the answer to the sub-questions of question three is negative) Do you think the noted fact creates discomfort for girls, especially during menstruation? Could the noted circumstance be a reason for missing school? Have you heard of a case when, because it was not possible to change in the school restroom, a student stayed home during menstruation? How would you assess the teachers' restroom in this regard?

If yes, how many such cases do you recall?

Does this have a systematic character?

In your assessment, did this affect the student's academic performance (grades or other assessment/encouragement)?
